# The association between healthcare needs, socioeconomic status, and life satisfaction from a Chinese rural population cohort, 2012–2018

**DOI:** 10.1038/s41598-022-18596-9

**Published:** 2022-08-19

**Authors:** Caiyun Chen, Richard Huan Xu, Eliza Lai-yi Wong, Dong Wang

**Affiliations:** 1 Nanjing Academy of Administration, Nanjing, China; 2 Party School of C.P.C. Nanjing Committee, Nanjing, China; 3 Department of Rehabilitation Sciences, The Hong Kong Polytechnic University, Hung Hom, Hong Kong SAR, China; 4 JC School of Public Health and Primary Care, The Chinese University of Hong Kong, Shatin, Hong Kong SAR, China; 5 School of Health Management, Southern Medical University, Guangzhou, China

**Keywords:** Health care, Risk factors

## Abstract

This study aimed to examine the prevalence of unmet healthcare needs and clarify its impact on socioeconomic status (SES) and life satisfaction in a longitudinal cohort of the Chinese rural population. Data used in this study were obtained from a nationally representative sample of 1387 eligible rural residents from the Chinese Family Panel Studies. Generalized estimating equation (GEE) logistic regression models were used to examine the factors associated with unmet healthcare needs and the impact of unmet healthcare needs on respondents’ perceived SES and life satisfaction. Approximately 34.6% of respondents were male, 18.2% were ≤ 40 years, and 66.7% had completed primary education or below. Around 19% and 32.6% of individuals who healthcare needs were met reported an above average socioeconomic status and life satisfaction, respectively in the baseline survey. GEE models demonstrated that unmet healthcare needs were significantly associated with low perceived SES (Odds ratio = 1.57, *p* < 0.001) and life satisfaction (Odds ratio = 1.23, *p* = 0.03) adjusted by covariates. Respondents who were older, reported moderate or severe illness, and with chronic conditions were more likely to report the unmet healthcare needs.Unmet healthcare needs are longitudinally associated with low SES and life satisfaction among the Chinese rural population, the disparity in access to healthcare exists among this population.

## Introduction

Equal access to healthcare is an important mechanism that mediates socioeconomic disparities in people’s health. A growing number of studies consistently show that people from higher socioeconomic groups are more likely to experience better health outcomes, report a lower risk of chronic conditions, and enjoy a longer life expectancy than those with lower socioeconomic status (SES)^1–3^. While looking specifically at rural populations, who have less utilization of or more unmet needs in regard to medical care, a similar pattern emerges ^4^. Despite significant policy attention and years of study, equal access to healthcare services in rural settings remains a health policy challenge^5^. There is increasing recognition that equal access to healthcare services can affect not only people’s health outcomes but also their social characteristics; moreover, it can create conditions that influence the configuration of local health- and social care systems^2,6^. However, in China, the long-term impact of exposure to unmet healthcare needs on perceived health development and social determinants of health and life satisfaction has rarely been reported in rural populations.

Providing and sustaining adequate and quality primary healthcare services for members of the Chinese rural population, who live under a dual-track healthcare system, is a major challenge that governments have faced for several decades^7^. With the rapid economic growth over the past three decades, the sociodemographic status and social structures in rural areas have changed significantly, which has resulted in widespread rural population decline, aging, and outmigration as well as prominent problems in the retention of medical professionals and the operation of locally available healthcare facilities. Although the reimbursement rate of the new cooperative medical scheme (NCMS) for the rural population is increasing, unmet healthcare needs remain, and their access to serviceable resources, including health literacy, financial support, and social connections, which affect their quality of life and social well-being, remain limited^8^.

In China, compared to urban residents, unequal access to quality health care is more prevalent among rural residents due to a high economic imbalance. In 2013, an updated NCMS was introduced by the central government, which aimed to greatly reduce rural patients’ financial burden of healthcare utilization through nearly full coverage^9^. However, the design and implementation of this NCMS varied across regions in China due to local socioeconomic conditions. Although with rapid urbanization, the wealth of the rural population has increased, with a growing number of them working in urban areas, given the strict hukou (family registry) system in China, they are still defined as rural residents, despite living in urban areas, and benefit only from limited social welfare schemes and services, compared to the “true” urban residents^10^. However, in 2016, the Chinese central government decided to combine the NCMS with urban resident medical schemes to reduce the urban–rural inequity in healthcare utilization. The modest reimbursement for inpatient and outpatient services has limited rural patients’ ability to use quality urban healthcare services^11^. According to 2018 China healthcare statistics, healthcare expenditure for urban residents paid by medical insurance is approximately twice that of rural residents^12^. This systematic inequity leads to the unmet healthcare needs of the rural population being higher than that of their urban counterparts due to lower financial protection, which is challenged by the implementation of rural revitalization strategies. However, empirical evidence regarding the threat of unmet healthcare needs to long-term health outcomes, equity, and social welfare in the Chinese rural population is limited. Thus, in this study, we used large state-level survey data to assess the relationship between unmet healthcare needs and perceived long-term SES and life satisfaction in a sample of the Chinese rural population.

## Method

### Data

The data used in this study were obtained from 2012, 2016, and 2018 online datasets of the Chinese Family Panel Studies (CFPS). The CFPS constitute a nationally representative and biannually longitudinal survey of communities, families, and individuals launched in 2010 with a focus on the economic state and well-being of the Chinese population. The information and dataset can be found on the CFPS official website (http://www.isss.pku.edu.cn/cfps/). The data used in this study were obtained via a formal application and were approved by the CFPS committee. Our sample was strictly restricted to individuals aged between 18 and 80 years as of the baseline survey and reported as rural residents according to their family register information at both the baseline and follow-up surveys.

### Measures

#### Demographic characteristics

Socioeconomic information regarding gender (male/female), age, educational attainment (primary or below/secondary or above), marital status (married/unmarried), employment status (active/non-active), and personal annual income (≤ 5000 RMB/5001–15000RMB/ ≥ 150001RMB) was collected.

#### Lifestyle and health status

Individuals’ lifestyle and health status information concerning chronic conditions (yes/no), body mass index (normal/abnormal), smoking and alcohol consumption in the previous six months (yes/no), type of visit to medical service delivery institutions (general outpatient/specialist/community center/clinics), and severity of illness (mild/moderate/severe) was collected.

#### Equity of access to and quality of healthcare services

Information about respondents’ agreement with the statement “equal needs have equal opportunities to access healthcare in China” was collected (1–10, where 10 indicated the most agreeable). The service quality was measured using the item “Are you satisfied with the last service provided by the medical professional?” and responded on a scale of 1 to 5. The results were categorized as bad (1–2), fair (3), and good (4–5) quality.

#### Perceived SES and life satisfaction

Perceived SES and life satisfaction were assessed using the following questions: “What do you think about your current SES in the local community?” and “What do you think about your current life satisfaction in the local community?” respectively. Respondents were asked to make their selection on a scale ranging from 0 to 5, with 0 indicating very low or very unsatisfied and five indicating very high or very satisfied. The outcome of these two items was regrouped as a binary categorical variable, which was equal to or below average and above average based on the mean score.

#### Healthcare needs

Unmet healthcare needs can be conceptualized as covering a spectrum of healthcare needs that are not optimally met. In this study, we defined unmet healthcare needs as an individual perceiving/reporting that they needed medical services but did not receive them^13^. Outcomes for two items (“Have you felt uncomfortable in the last two weeks?” [yes/no] and “Have you visited doctors?” [yes/no]) were used to measure healthcare needs. Eligible respondents were restricted to individuals who reported feeling uncomfortable (those who selected YES on the first item) in the last two weeks in all three rounds of the survey. Subsequently, individuals who reported not visiting doctors (those who selected NO on the second item) were defined as respondents with unmet healthcare needs and vice versa (those who selected YES were defined as those whose healthcare needs were met).

#### Depressive status

The Center for Epidemiologic Studies-Depression Scale (CES-D) was used to assess depressive symptoms^14^. The CES-D scale is a 20-item instrument with each item rated on a four-point scale ranging from 0 (“rarely or none of the time”) to 3 (“most or all of the time”) reflecting the frequency of depressive symptoms. The threshold for detecting depression on the CES-D was identified as 16 (range: 0–60)^15^.

### Statistical analysis

Descriptive analysis was used to describe respondents’ demographics, lifestyle, and health status as proportions and percentages. Chi-square tests were used to examine the demographics, lifestyle, and health status of respondents with different statuses of illness at the baseline survey. We also used chi-square tests to examine the differences in severity of illness, perceived SES, life satisfaction, service quality, and type of visited medical service delivery institutions between the three rounds of the survey. The Kruskal–Wallis test and Wilcoxon signed-rank test were used to assess the differences between respondents’ attitudes toward equality of access to healthcare services and severity of illness. To analyze the longitudinal relationship between healthcare needs, perceived SES, and life satisfaction, we adopted a generalized estimating equation (GEE) logistic regression model, using a robust sandwich variance estimator^16^. GEE models are an extension of generalized linear models and widely used for analyzing longitudinal data^17,18^. It uses all available longitudinal data, allows unequal numbers of repeated measurements, and has some robustness against deviation from normality. Measure for the healthcare needs and all the covariates are repeated measures, except for several time-invariant variables (e.g., sex). Our GEE models included healthcare needs (dependent variable), SES, life satisfaction, demographics, lifestyle, depressive status, and severity of illness. Odds ratios (ORs) and 95% confidence intervals (95% CIs) from GEE logistic models were estimated to predict the probabilities of perceived SES and life satisfaction adjusted for respondents’ background characteristics. Analyses were performed using the R software, and *p* values < 0.05 were considered statistically significant.

### Ethical approval and consent to participate

All study participants gave informed consent to participate in the original survey, and the study was conducted according to the principles embodied in the Declaration of Helsinki. Ethical approval of this analysis was granted by the Institutional Review Board of the Hong Kong Polytechnic University (Ref: ﻿HSEARS20210714009).

## Results

Data from 1,387 rural residents who met our criteria were included in the analysis. Among them, 476 (34.6%) were male, 249 were ≤ 40 years (18.2%), and 926 only completed primary education or below (66.7%). Regarding the severity of illness, respondents who were young, highly educated, and actively employed, had no chronic conditions, and were well paid, reported a significantly higher proportion of mild illness (Table 1).

**Table 1:** General characteristics and stratified by disease severity group at baseline in 2012, n (%) 2012, China.

	Disease status					
	Overall (n = 1387)	Mild (n = 222)	Moderate (n = 513)	Severe (n = 652)	t/ x2 (d.f.)	*p*
**Sex**						
Male	476 (34.6)	74 (33.8)	179 (35.2)	223 (34.4)	0.2 (2)	0.93
Female	900 (65.4)	145 (66.2)	330 (64.8)	425 (65.6)		
**Age**						
≤ 40 years	249 (18.2)	48 (22.2)	124 (24.5)	77 (11.9)	40.6 (6)	< 0.001
41 ~ 50 years	416 (30.4)	63 (29.2)	158 (31.2)	195 (30.2)		
51 ~ 60 years	425 (31.1)	71 (32.9)	129 (25.5)	225 (34.8)		
≥ 60 years	278 (20.3)	34 (15.7)	95 (18.8)	149 (23.1)		
**Educational level**						
Primary or below	926 (66.7)	130 (65)	316 (69.8)	480 (78.7)	19 (2)	< 0.001
Secondary or above	337 (33.3)	70 (35)	137 (30.2)	130 (21.3)		
**Married status**						
Married	1132 (81.6)	173 (86.5)	411 (90.5)	548 (89.8)	2.5 (2)	0.29
Unmarried	132 (9.5)	27 (13.5)	43 (9.5)	62 (10.2)		
**Employed status**						
Actively	791 (57)	131 (60.1)	310 (60.9)	350 (53.8)	6.6 (2)	0.04
Non-actively	586 (42.2)	87 (39.9)	199 (39.1)	300 (46.2)		
**Chronic condition**						
Yes	343 (24.7)	33 (14.9)	119 (23.2)	191 (29.3)	19.2 (2)	< 0.001
No	1044 (75.3)	189 (85.1)	394 (76.8)	461 (70.7)		
**Personal annual income**						
≤ 5000RMB	1155 (83.3)	175 (78.8)	402 (78.4)	578 (88.8)	31.9 (4)	< 0.001
5001-15000RMB	128 (9.2)	20 (9)	61 (11.9)	47 (7.2)		
≥ 150001RMB	103 (7.4)	27 (12.2)	50 (9.7)	26 (4)		
**Body mass index**						
Normal	769 (60.1)	127 (59.3)	293 (62.5)	349 (58.6)	1.7 (2)	0.42
Unnormal	510 (39.9)	87 (40.7)	176 (37.5)	247 (41.4)		
**Smoke**						
No	1055 (76.1)	169 (76.1)	399 (77.8)	487 (74.7)	1.5 (2)	0.47
Yes	332 (23.9)	53 (23.9)	114 (22.2)	165 (25.3)		
**Alcohol consumption**						
No	1244 (89.7)	197 (88.7)	460 (89.7)	587 (90)	0.3 (2)	0.86
Yes	143 (10.3)	25 (11.3)	53 (10.3)	65 (10)		

**Figure 1:**
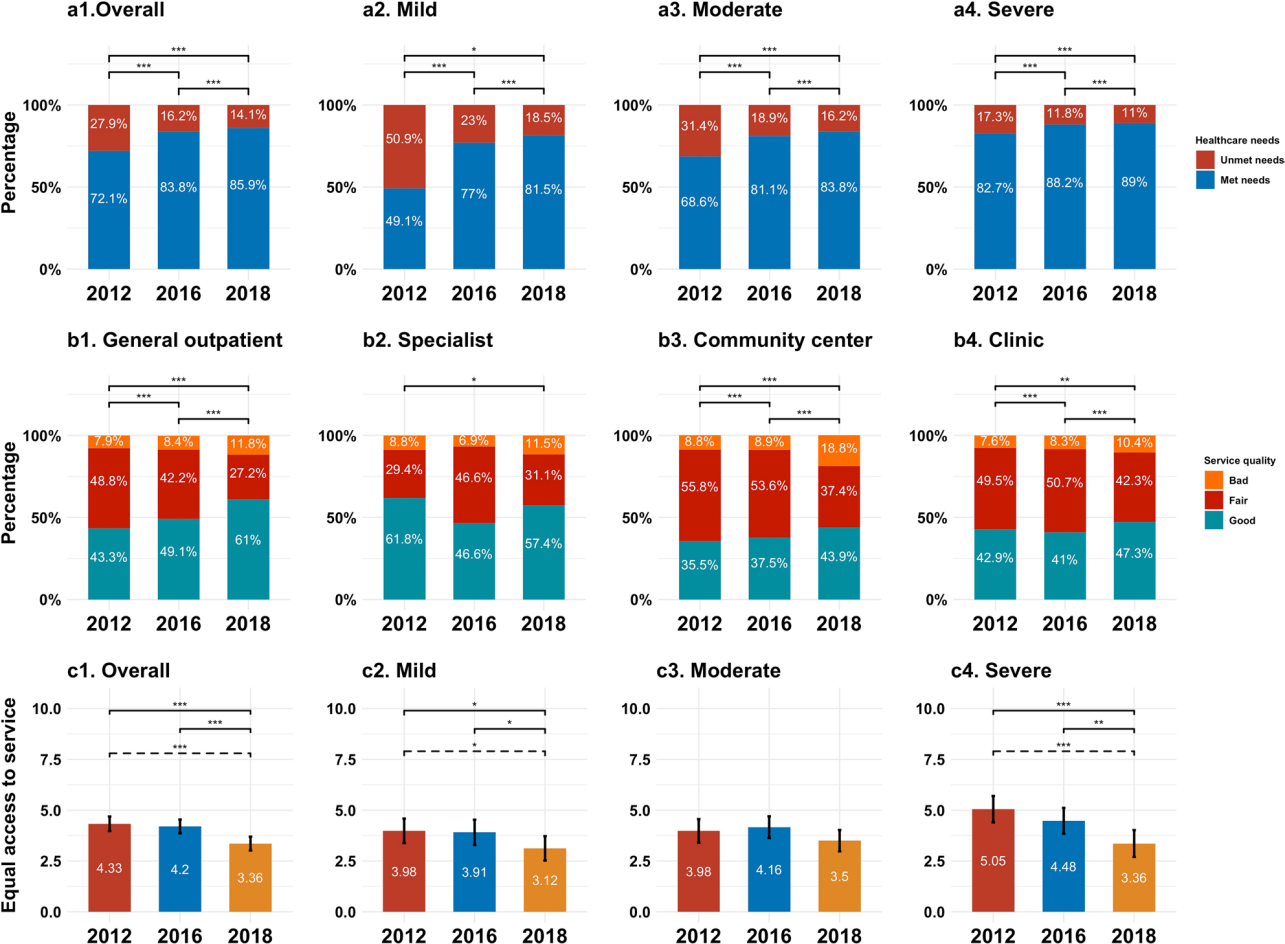
Association between unmet healthcare needs, severity of illness, quality of service and equal access to service, 2012–2018, China.

Table 2 demonstrates that a higher proportion of respondents who showed a low perceived SES and life satisfaction were more likely to report unmet healthcare needs in the baseline survey and follow-ups. We found that more respondents whose healthcare needs were met reported a higher SES in all three surveys, regardless of their depressive status. Only respondents who showed no depressive symptoms and had unmet healthcare needs exhibited significantly lower life satisfaction than those who reported that their healthcare needs were met in the 2018 survey. Regarding the relationship between severity of illness and healthcare needs, the overall proportion of unmet healthcare needs decreased from 27.9% in 2012 to 14.1% in 2018. Respondents with mild illness reported the largest decrease rate of 32.4% from 2012 to 2018 (part a, Fig. 1).

**Table 2:** Relationship between health care needs, SES and life satisfaction and stratified by depressive status in different follow-ups, n (%).

	2012			2016			2018		
	Unmet need	Met need	*p*	Unmet need	Met need	*p*	Unmet needs	Met needs	*p*
**Overall**									
Socioeconomic status									
Below or equal to average	330 (86.6)	797 (81)	0.01	187 (83.5)	826 (71.6)	< 0.001	150 (76.9)	700 (59)	< 0.001
Above average	51 (13.4)	187 (19)		37 (16.5)	328 (28.4)		45 (23.1)	487 (41)	
Life satisfaction									
Below or equal to average	269 (70.1)	670 (67.4)	0.34	131 (58.2)	590 (50.9)	0.05	90 (45.9)	414 (34.8)	0.002
Above average	115 (29.9)	324 (32.6)		94 (41.8)	568 (49.1)		106 (54.1)	777 (65.2)	
**Depressive status**									
Socioeconomic status									
Below or equal to average	162 (88.5)	408 (80.5)	0.01	96 (85.7)	491 (75.1)	0.01	84 (80.8)	371 (62.5)	< 0.001
Above average	21 (11.5)	99 (19.5)		16 (14.3)	163 (24.9)		20 (19.2)	223 (37.5)	
Life satisfaction									
Below or equal to average	138 (75)	372 (72.8)	0.56	74 (9)65.5	377 (57.5)	0.1	53 (51)	255 (42.9)	0.12
Above average	46 (25)	139 (27.2)		39 (34.5)	279 (42.5)		51 (49)	340 (57.1)	
**Non-depressive status**									
Socioeconomic status									
Below or equal to average	160 (86.5)	356 (82)	0.17	91 (82)	332 (66.8)	0.002	64 (71.9)	324 (55.3)	0.003
Above average	25 (13.5)	78 (18)		20 (18)	165 (33.2)		25 (28.1)	262 (44.7)	
Life satisfaction									
Below or equal to average	120 (64.2)	263 (60.7)	0.47	57 (51.4)	212 (42.4)	0.08	35 (38.9)	156 (26.6)	0.02
Above average	67 (35.8)	170 (39.9)		54 (48.6)	288 (57.6)		55 (61.1)	431 (73.4)

Figure 1 (part b) shows the respondents’ ratings of the service quality of different medical service delivery institutions. Overall, fewer respondents were satisfied with the service quality of community healthcare centers than were those visiting other medical service delivery institutions. Respondents using general outpatient services reported the largest increase in the rate of satisfaction with service quality, from 43.3% in 2012 to 61% in 2018, compared to those using the other types of services. The rate of satisfaction with service quality for specialist services dropped from 61.8% in 2012 to 57.4% in 2018. Figure 1 (part c) demonstrates that respondents with unmet healthcare needs indicated a significant decrease in agreement with having equal access to healthcare services from 2012 (mean = 4.33/10) to 2018 (mean = 3.36/10).

Table 3 shows the results of the GEE models conducted separately to predict the associations between (1) healthcare needs and respondents’ background characteristics (Model 1) and (2) healthcare needs and perceived SES and life satisfaction (Models 2 and 3), respectively. In Models 2 and 3, potential confounders (i.e., respondents’ demographics, health status, and lifestyles) were adjusted. The ORs and 95% CI showed that there was a significant association between unmet healthcare needs and low perceived SES (OR = 1.56, 95% CI = 1.28, 1.92) and life satisfaction (OR = 1.23, 95% CI = 1.02, 1.48). Older respondents, with moderate or severe illness, and with comorbid chronic conditions were more likely to report unmet healthcare needs.

**Table 3:** Odds ratios (95% confidence intervals) for associations between healthcare needs and social status and life satisfaction.

	Model 1^#^			Model 2			Model 3		
	OR	95%C.I	*p*	OR	95% C.I	*p*	OR	95% C.I	*p*
**Socioeconomic status**									
Above average				Ref					
Below or equal to average				1.56	1.26,1.93	< 0.001			
**Life satisfaction**									
Above average							Ref		
Below or equal to average							1.23	1.05,1.48	0.02
**Sex**									
Female	Ref			Ref			Ref		
Male	0.99	0.76,1.3	0.95	1.01	0.54,1.32	0.92	0.98	0.77,1.26	0.94
**Age**									
≤ 40 years	Ref			Ref			Ref		
41 ~ 50 years	1.68	1.31,2.16	< 0.001	1.62	1.23,1.87	< 0.001	1.67	1.13,1.82	< 0.001
51 ~ 60 years	1.82	1.42,2.34	< 0.001	1.71	1.23,2.31	< 0.001	1.77	1.51,2.21	< 0.001
≥ 60 years	1.98	1.45,2.66	< 0.001	1.76	1.55,3.33	< 0.001	1.89	1.66, 3.3	< 0.001
**Education**									
Primary or below	Ref			Ref			Ref		
Secondary or above	1.06	0.87,1.28	0.55	1.05	0.19,1.26	0.64	1.04	0.6,1.66	0.69
**Severity of illness**									
Mild	Ref			Ref			Ref		
Moderate	1.52	1.2,1.92	< 0.001	1.52	1.2,1.99	< 0.001	1.54	1.21,1.54	< 0.001
Severe	2.48	1.95,3.13	< 0.001	2.41	1.71,3.63	< 0.001	2.5	2.2,3.31	< 0.001
**Depression**									
No	Ref			Ref			Ref		
Yes	0.91	0.76,1.08	0.33	0.93	0.45,1.19	0.45	0.93	0.17,0.67	0.45
**Employment**									
Non-actively	Ref			Ref			Ref		
Actively	0.99	0.82,1.16	0.87	0.97	0.86,1.2	0.83	0.98	0.39,1.16	0.84
**Income**									
≤ 5000RMB	Ref			Ref			Ref		
5001-15000RMB	0.83	0.63,1.12	0.21	0.85	0.11,1.91	0.29	0.84	0.1,0.97	0.25
≥ 150001RMB	0.78	0.56,1.07	0.15	0.77	0.09,1.87	0.15	0.78	0.03,1.45	0.16
**Chronic condition**									
No	Ref			Ref			Ref		
Yes	1.42	1.12,1.79	0.003	1.42	1.12,2.32	0.002	1.43	1.06,2.1	0.002
**BMI**									
Unnormal	Ref			Ref			Ref		
Normal	1.13	0.94,1.34	0.2	1.12	0.71,1.57	0.23	1.12	0.91,1.66	0.2
**Smoke**									
No	Ref			Ref			Ref		
Yes	0.83	0.63,1.08	0.16	0.83	0.03,1.42	0.17	0.83	0.61,1.98	0.19
**Alcohol consumption**									
No	Ref			Ref			Ref		
Yes	0.84	0.63,1.13	0.25	0.82	0.09,1.57	0.18	0.83	0.03,1.54	0.22

^#^Reference = healthcare needs met.

## Discussion

This study demonstrates that higher unmet healthcare needs were longitudinally associated with lower perceived SES and life satisfaction in the rural Chinese population. Healthcare needs are an independent predictor of long-term SES, even after adjusting for individuals’ sociodemographic characteristics, health status, and lifestyle. The outcomes of the logistic regression model confirmed that older rural residents, with moderate or worse illness, and with comorbid chronic conditions were more likely to report having significant unmet healthcare needs. Our findings suggest that healthcare needs involve important elements of individuals’ social well-being and health equity, a fact that has not received adequate attention in previous SES studies of rural populations. Given that the concepts of perceived SES and life satisfaction in this study were subjective, respondents’ mental well-being was also considered in our analysis and showed a significant impact on the effect of unmet healthcare needs on respondents’ SES. Mental health problems are associated with chronic physical disease and socioeconomic disadvantage in China^19,20^, and the utilization of mental health services in rural areas is insufficient, compared to that in urban areas^21^. We suggest that unequal access to healthcare is not only a health issue but a social issue and postulate that it may increase social disparities within communities, between regions, and even across generations. Future research will be needed to examine these mechanisms and develop possible interventions to reduce health-related social inequality.

Met/unmet healthcare needs have been increasingly identified as a critical indicator of access to care within and across different healthcare systems^22,23^. However, the outcomes of evaluation can vary owing to the different definitions, settings, or populations of healthcare needs. In this study, we referred to healthcare needs as the difference between healthcare services deemed necessary to manage a particular health problem and actual services received, which is in line with several previous studies^24,25^. However, as health is increasingly seen by the public health community as a multidimensional construct that includes physical, mental, and social domains^26^, the connotations of healthcare needs change and broaden accordingly. The significant association between unmet healthcare needs and low perceived SES and life satisfaction can be reasonably explained if we recognize healthcare needs as the health aspect of broader social care needs, which aim to provide individuals with assistance with activities of daily living, to maintain their independence, and to enable them to better engage in social activities^27^. It has been confirmed that long-term unmet needs are more likely to lead to mental health problems^28^, readmission^29^, and high mortality rates^30^, which, in turn, lower individuals’ SES and life satisfaction. Additionally, considering that China has a rural–urban dual system in which socioeconomic disparity is substantial and urbanization has been rapid in recent decades, the divide between rural and urban areas in terms of demographic structure, social norms, and economic development has widened. Reducing unmet healthcare needs is important to bridge the boundaries among social, mental, and medical services for rural populations.

Social class has been confirmed by previous studies as a fundamental determinant of health, and the association is believed to cross-cultural and geographic boundaries. A close relationship between unmet healthcare needs and individuals’ SES has been reported in several previous studies. For example, people who were females, ethnic minorities, low income, or had chronic conditions were more likely to report a high possibility of having the unmet healthcare needs^31–34^. However, our data revealed that individuals reporting high unmet needs were those who reported living with mild illness. Further analyses also showed that individuals with unmet healthcare needs were highly likely to have unequal access to medical services. This phenomenon revealed that rural residents who did not consistently use healthcare services may have failed to do so not because of their mild illness but because they were unable to access affordable quality medical services, which is partially in line with previous findings^35–37^ but has never been discussed in terms of the Chinese rural population. Further studies should explore which factors contribute to unequal access to medical care, quantify their associations with unmet healthcare needs, and investigate their longitudinally detrimental effect on rural residents’ SES.

One interesting finding is that increasing input in rural healthcare facilities in recent decades does not seem to have led to commensurate improvements in rural residents’ life satisfaction. Life satisfaction is identified as a subjective concept and is difficult to measure. A previous study on life satisfaction indicated that simply assuming fast economic growth can lead to a happy population is not accurate^38^. This study demonstrated a causal relationship between unmet healthcare needs and low life satisfaction in a rural population in China. China has experienced rapid and substantial economic growth for more than 30 years, and the NCMS provides health insurance coverage to the rural population, which has greatly changed rural people’s health over time^39^. However, a recent study found that Chinese rural residents from low-income groups still report a high financial risk^40^, which leads to a high rate of unmet healthcare needs. Moreover, we found that despite the enhanced satisfaction with the quality of healthcare services, an increased proportion of respondents reported dissatisfaction with the quality of healthcare services for all types of medical services from 2012 to 2018. This indicates that despite the existence of a causal relationship between unmet healthcare needs and life satisfaction, the mediating effect of the quality of healthcare services should be further investigated. The NCMS should gradually transfer its focus from increasing population coverage to improving service coverage and reimbursement rates. Rural residents are the most challenging population for whom to improve health outcomes due to their low income, poor health status, and limited access to health services^41^. Our findings confirmed that unmet healthcare needs can be a detrimental factor leading to socioeconomic disadvantages and life dissatisfaction in the rural Chinese population, and its impact is consistent and far-reaching.

This study had several limitations. First, although our sample elicited from a nationally representative sample, only data from around 1300 individuals, who met our study purpose, were elicited for analysis, which may have led to selection bias and affected the generalizability of our findings. Second, information regarding SES and life satisfaction was self-reported, which may have led to recall bias. Third, even if respondents reported the rural family registry in both 2012 and 2018, they may have been living in an urban rather than a rural area, and we cannot differentiate between these two groups in this study, which may have led to selection bias.

## Conclusions

This study demonstrated that unmet healthcare needs are longitudinally associated with low SES and life satisfaction among the Chinese rural population, and the disparity in access to healthcare services led to significant inequity in service utilization. This study also showed that advanced age, severe illness, and comorbid chronic conditions were factors that increased rural residents’ unmet healthcare needs. In 2016, the Chinese government decided to combine urban and rural resident medical insurance schemes to reform the cost-sharing mechanism. This may prove to be a game-changer for reducing healthcare disparity between urban and rural areas; however, its effects may not be measurable from our data. Future studies are needed to investigate its impact on rural populations’ long-term SES and life satisfaction.

## Supplementary Information

Supplementary Information.

## Data Availability

The data that support the findings of this study are available from the Chinese Family Panel Studies but restrictions apply to the availability of these data, which were used under license for the current study, and so are not publicly available. Data are however available from the authors upon reasonable request and with permission of the Chinese Family Panel Studies.
