# Supplementary material for: The association between healthcare needs, socioeconomic status, and life satisfaction from a Chinese rural population cohort, 2012–2018
Source: Sci Rep. 2022 Aug 19;12:14129. doi: 10.1038/s41598-022-18596-9 (PMC9391494; doi:10.1038/s41598-022-18596-9)
Supplement: Supplementary file 1 — Supplementary Information. [file 41598_2022_18596_MOESM1_ESM.docx]

Table S.1 Comparisons between sample and total population at baseline (2012)

| Sex | Sample | Overall rural population  (n=25,816) | p-value |
| --- | --- | --- | --- |
| Male | 476 (34.6) | 12420(49.9) | <0.001 |
| Female | 900(65.4) | 12471(50.1) |  |
| Age |  |  |  |
| ≤ 40 years | 249(18.2) | 11045(45.3) | <0.001 |
| 41~50 years | 416(30.4) | 5433(22.3) |  |
| 51~60 years | 425(31.1) | 4074(16.7) |  |
| ≥ 60 years | 278(20.3) | 3845(15.8) |  |
| Educational level |  |  |  |
| Primary or below | 926(66.7) | 11637(61.1) | <0.001 |
| Secondary or above | 337(33.3) | 7409(38.9) |  |
| Married status |  |  |  |
| Married | 1132(89.6) | 14960(80.3) | <0.001 |
| Unmarried | 132(10.4) | 3664(19.7) |  |
| Employed status |  |  |  |
| Actively | 791(57) | 14360(58.7) | 0.35 |
| Non-actively | 586(42.2) | 10087(41.3) |  |
| Chronic condition |  |  |  |
| Yes | 343(24.7) | 2561(11.2) | <0.001 |
| No | 1044(75.3) | 20353(88.8) |  |
| Personal annual income |  |  |  |
| ≤5000RMB | 1155(83.3) | 17447(67.7) | <0.001 |
| 5001-15000RMB | 128(9.2) | 3045(11.8) |  |
| ≥150001RMB | 103(7.4) | 5279(20.5) |  |
| Body mass index |  |  |  |
| Normal | 769(60.1) | 13565(60.9) | 0.62 |
| Unnormal | 510(39.9) | 8726(39.1) |  |
| Smoke |  |  |  |
| No | 1055(76.1) | 16109(70.3) | <0.001 |
| Yes | 332(23.9) | 6814(29.7) |  |
| Alcohol consumption |  |  |  |
| No | 1244(89.7) | 19390(84.6) | <0.001 |
| Yes | 143(10.3) | 3533(15.4) |  |
